# Bacterial natural product discovery by heterologous expression

**DOI:** 10.1093/jimb/kuad044

**Published:** 2023-12-05

**Authors:** Adjo E Kadjo, Alessandra S Eustáquio

**Affiliations:** Department of Pharmaceutical Sciences, College of Pharmacy, University of Illinois at Chicago, Chicago, IL 60607, USA; Center for Biomolecular Sciences, College of Pharmacy, University of Illinois at Chicago, Chicago, IL 60607, USA; Department of Pharmaceutical Sciences, College of Pharmacy, University of Illinois at Chicago, Chicago, IL 60607, USA; Center for Biomolecular Sciences, College of Pharmacy, University of Illinois at Chicago, Chicago, IL 60607, USA

**Keywords:** Heterologous expression, Bacteria, Metabolites, Natural products, Synthetic biology

## Abstract

Natural products have found important applications in the pharmaceutical and agricultural sectors. In bacteria, the genes that encode the biosynthesis of natural products are often colocalized in the genome, forming biosynthetic gene clusters. It has been predicted that only 3% of natural products encoded in bacterial genomes have been discovered thus far, in part because gene clusters may be poorly expressed under laboratory conditions. Heterologous expression can help convert bioinformatics predictions into products. However, challenges remain, such as gene cluster prioritization, cloning of the complete gene cluster, high level expression, product identification, and isolation of products in practical yields. Here we reviewed the literature from the past 5 years (January 2018 to June 2023) to identify studies that discovered natural products by heterologous expression. From the 50 studies identified, we present analyses of the rationale for gene cluster prioritization, cloning methods, biosynthetic class, source taxa, and host choice. Combined, the 50 studies led to the discovery of 63 new families of natural products, supporting heterologous expression as a promising way to access novel chemistry. However, the success rate of natural product detection varied from 11% to 32% based on four large-scale studies that were part of the reviewed literature. The low success rate makes it apparent that much remains to be improved. The potential reasons for failure and points to be considered to improve the chances of success are discussed.

**One-Sentence Summary:**

At least 63 new families of bacterial natural products were discovered using heterologous expression in the last 5 years, supporting heterologous expression as a promising way to access novel chemistry; however, the success rate is low (11–32%) making it apparent that much remains to be improved—we discuss the potential reasons for failure and points to be considered to improve the chances of success. BioRender was used to generate the graphical abstract figure.

## Graphical Abstract

**Graphical Abstract:**
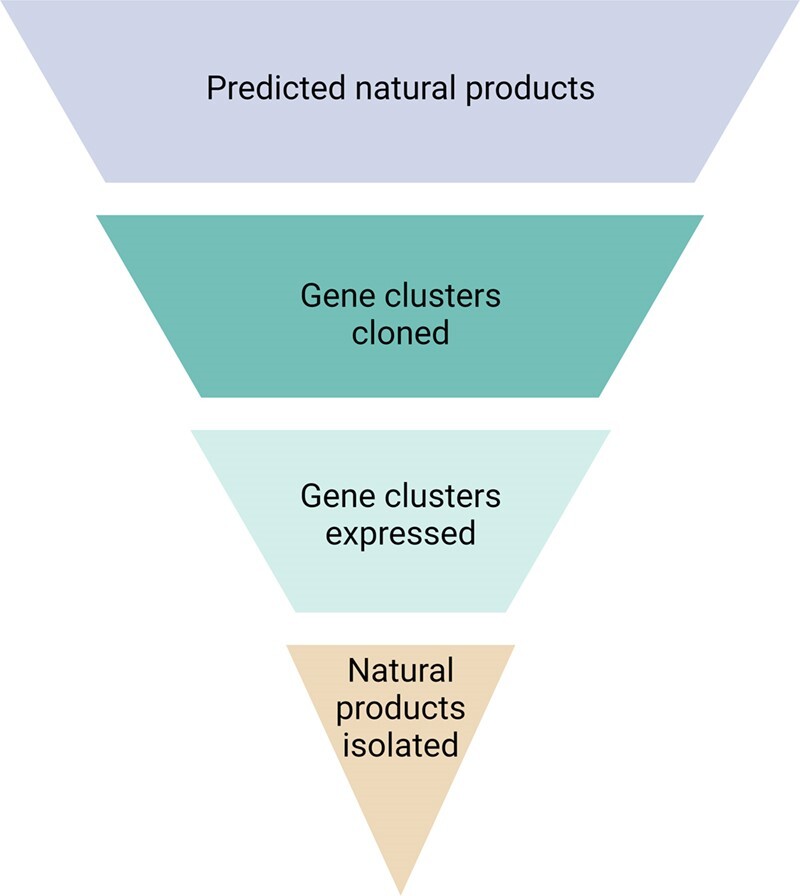

## Introduction

Natural products play important roles in the agricultural and pharmaceutical sectors. For instance, most small molecule drugs approved by the US Food and Drug Administration are either natural product (NP), NP derivatives, or synthetic compounds with NP pharmacophores (Newman & Cragg, 2020). To counteract drug resistance, it is necessary to discover new compounds. Additionally, it has been estimated that 85% of the human, disease-associated proteome lack an associated therapeutic (Neklesa et al., 2017), implying a large therapeutic gap that could be at least partially filled by NP discovery.

Advances in DNA sequencing and bioinformatics have revealed the untapped NP biosynthesis potential of microorganisms (Bachmann et al., 2014; Gavriilidou et al., 2022). A recent study predicted that only 3% of NPs encoded in bacterial genomes have been discovered (Gavriilidou et al., 2022). The discovery of new NPs is hampered by two main factors. First, most microorganisms from which NPs can be discovered remain uncultured. Second, the conditions used in the laboratory to study culturable microorganisms may not be appropriate for production in amounts that enable discovery and development (Baltz, 2017).

In bacteria, the genes that encode the biosynthesis of a NP are often colocalized in the genome, forming biosynthetic gene clusters (BGCs). Most BGCs have not been connected to a NP and are thus termed orphan. The exploration of orphan BGCs in the native producers or through heterologous expression offers an avenue for discovery. Strategies that have been used to activate gene expression and access the biosynthetic potential of microorganisms in the native producers include variation of the culture conditions, addition of elicitors to the culture media, co-cultivation to replicate the environmental conditions that promote NP production, and genetic approaches as reviewed here (Covington et al., 2021). Genetic approaches can be targeted to a specific NP of interest. Examples include deletion of pathway-specific negative regulators, overexpression of positive regulators, or promoter exchange (Covington et al., 2021). A drawback of genetic engineering of native producers is that tools for genetic manipulation must be developed for each strain of interest. Moreover, native producer centric methods cannot be used for uncultured bacteria.

**Fig. 1.**
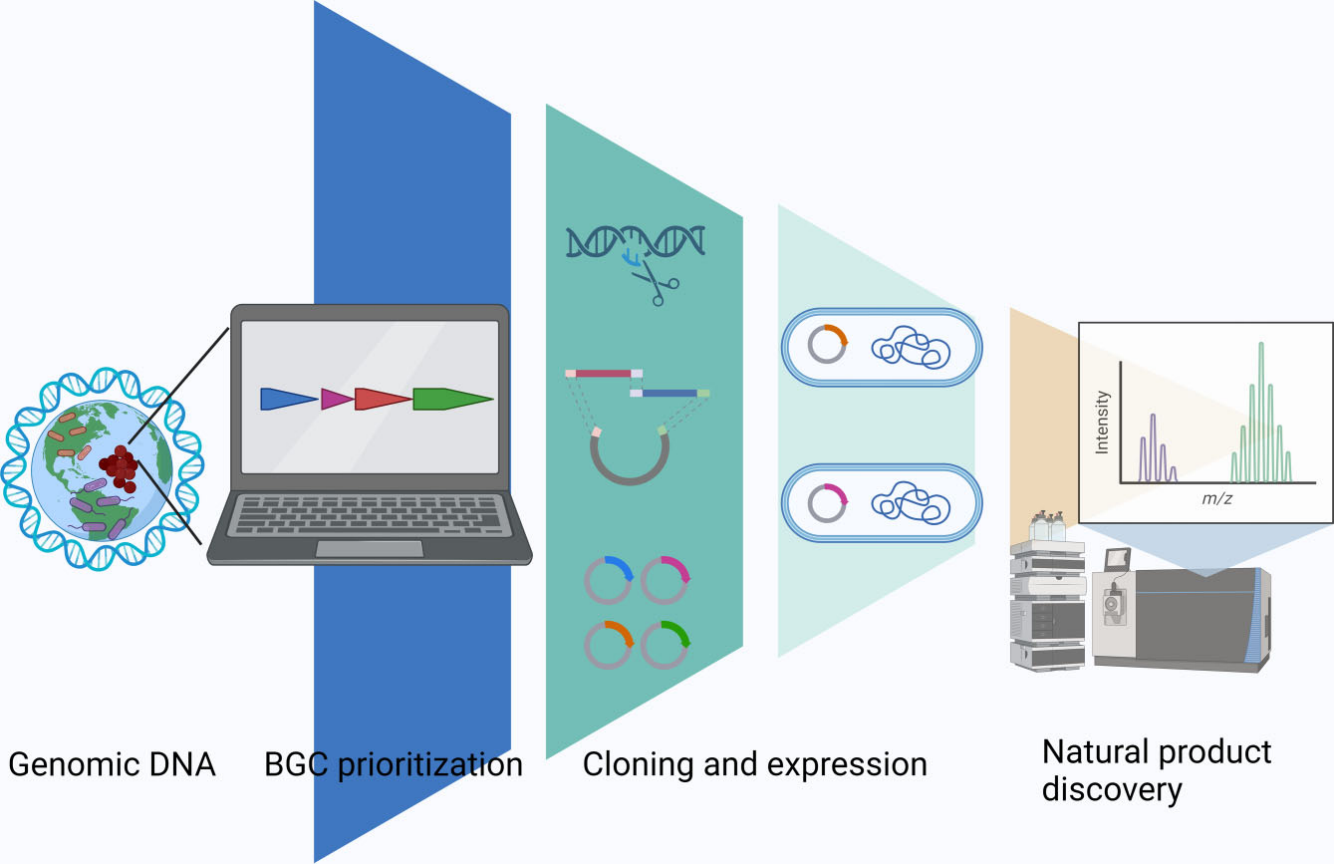
Pipeline for natural product discovery by heterologous expression. Genomic DNA is sequenced and analyzed with bioinformatic tools. Biosynthetic gene clusters (BGCs) are predicted and prioritized. The selected BGCs are cloned and transferred into a suitable host for expression. Metabolomics tools are used for natural product detection, and if enough quantities are produced, the natural product is isolated and characterized. BioRender was used to generate this figure.

Alternatively, heterologous expression of orphan BGCs in an established host strain offers great potential for NP discovery (Fig. 1). BGCs that are not well expressed under laboratory conditions can be refactored for activation and NPs from uncultured bacteria or metagenomes can be explored as well. However, some major challenges are BGC prioritization, cloning of the complete BGC, appropriate expression of the BGC in the selected host, and the identification and isolation of products in practical yields.

**Fig. 2.**
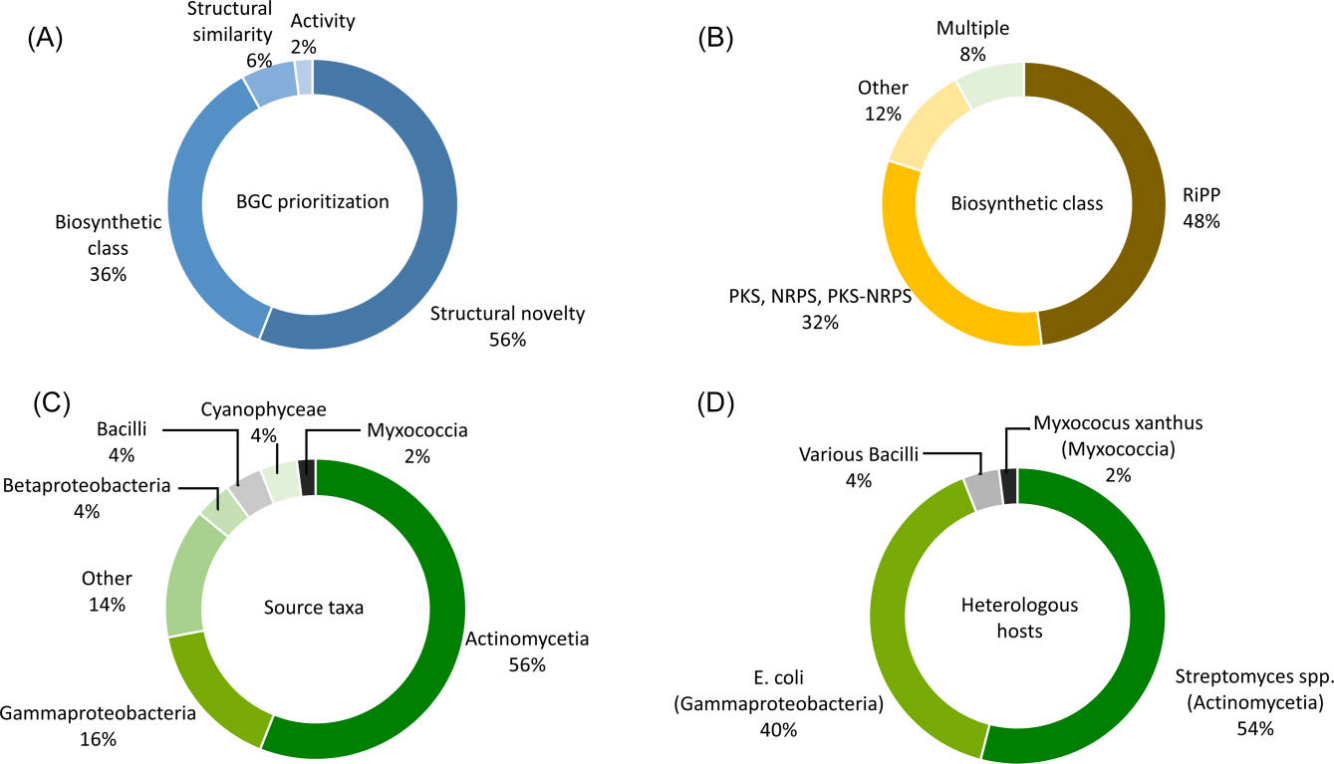
Discovery of bacterial natural products by heterologous expression in the last 5 years. The 50 studies reviewed here are broken down by (**A**) Rationale for BGC prioritization. (**B**) Biosynthetic class of prioritized BGCs. (**C**) Source of BGCs. (**D**) Heterologous hosts used.

**Fig. 3.**
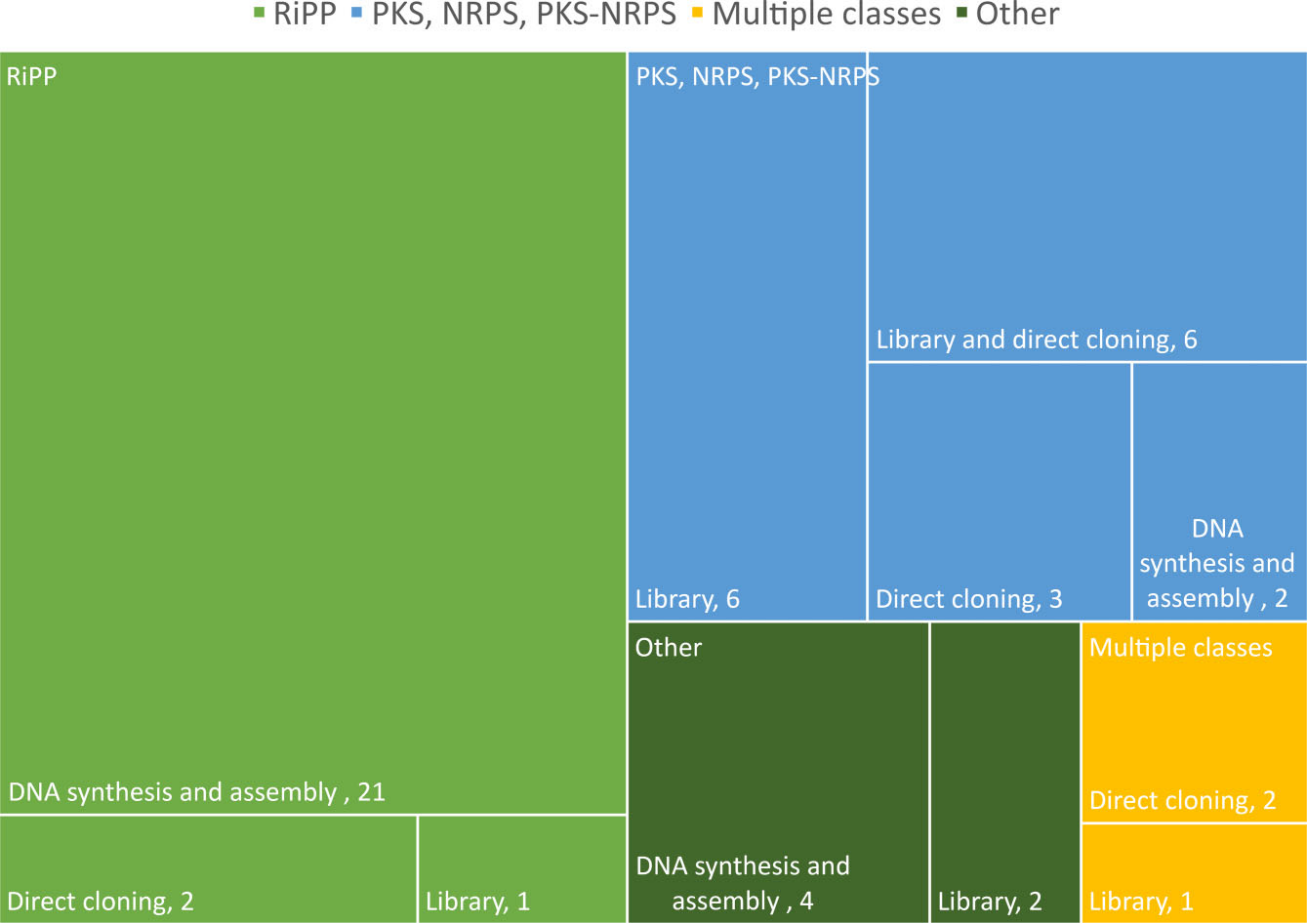
Cloning techniques used for different BGC classes. DNA synthesis refers to DNA synthesis or PCR. Assembly methods include restriction enzymes mediated methods and recombination-based methods. Random libraries were generated using cosmid or BAC vectors.

**Fig. 4.**
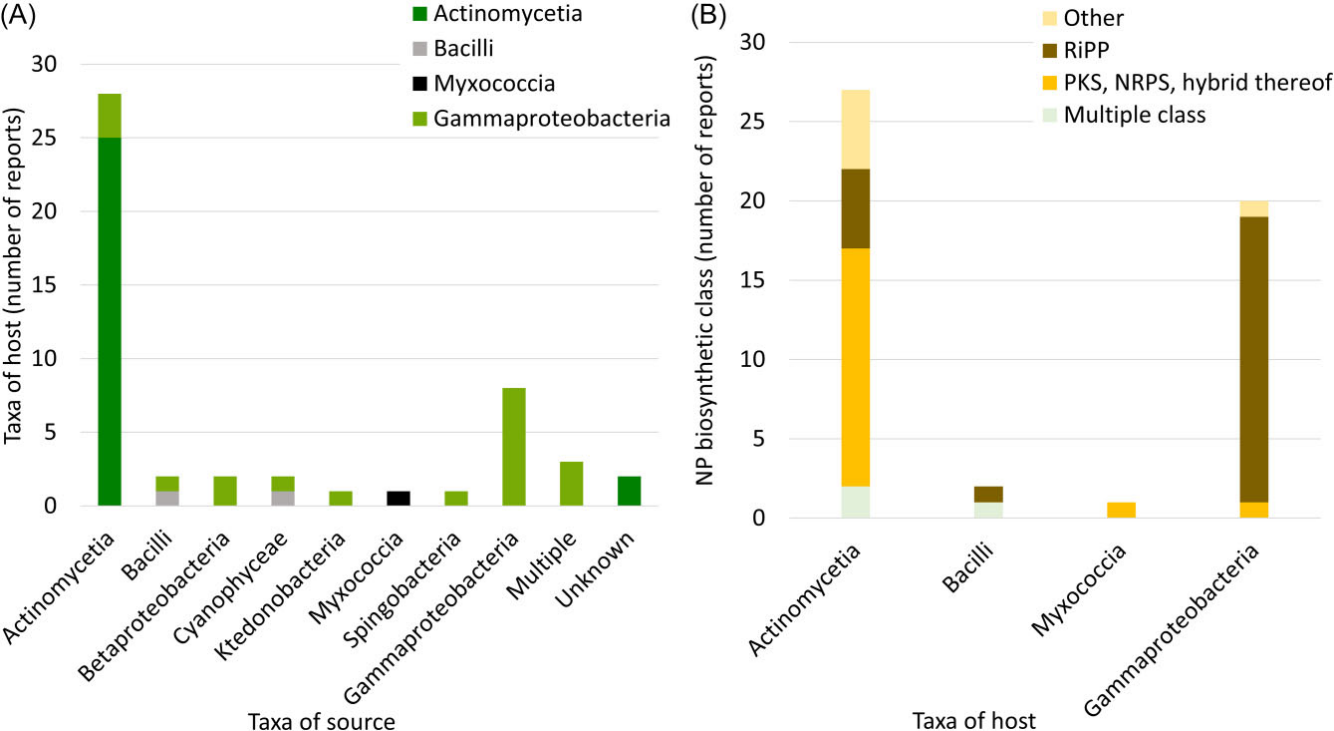
Metrics regarding source taxa, host taxa, and biosynthetic class. (**A**) Relationship between the taxa of the BGC source (*x-*axis) and the taxa of the heterologous host (*y*-axis). Studies that tested more than one source taxa were labeled as multiple. Host taxa (class) are color-coded as indicated. (**B**) Relationship between the taxa of the host used (*x-*axis) and the biosynthetic class of the expressed BGC (*y*-axis). NP biosynthetic classes are color coded as indicated.

To provide insight on what has worked and potential causes of heterologous expression failure, we searched PubMed and Web of Science for articles published between January 2018 and June 2023 using search terms ‘natural product’ AND ‘heterologous expression’ AND ‘bacteria’. We also searched with the terms ‘biosynthetic gene cluster’ AND ‘heterologous expression’ AND ‘bacteria’ AND ‘discovery’. Additionally, we used the search terms ‘genome mining’ AND ‘heterologous expression’ AND ‘bacteria’. Because our aim was to focus the review on heterologous expression for NP discovery, studies were excluded if they only reported rediscovery of known NPs, close congeners of known NPs, or NPs previously detected in native producers. Despite careful analysis, we expect we may have missed relevant articles that were not identified using the search terms above and apologize to researchers whose work we inadvertently omitted. Based on the 50 identified articles (Supplemental Table S1), below we discuss the rationale for BGC prioritization, cloning methods, biosynthetic class, source taxa, and host choice (Figs 2–4). We then summarize and discuss large scale studies that have allowed the determination of success rates (Table 1). We conclude with a discussion of remaining challenges. Our goal is to obtain insights on approaches used with the hope of informing researchers whose goal is to find natural products from orphan BGCs.

**Table 1.** Summary of large scale, heterologous expression studies to discover natural products.

BGC source	No. of BGCs selected for cloning	No. of BGCs cloned (success rate)	Biosynthetic class	BGC/insert size (kb)	Cloning method	Host(s) used	No. of BGCs expressed (success rate)	No. of NP families isolated	Ref.
1 *Saccharothrix espanaensis*	25	17 (68%)	Multiple	100	Random library	*S. lividans* DYA *S. albus* J1074	4 (11%)	2	(Gummerlich et al., 2020)
14 *Streptomyces spp.* 3 *Bacillus spp*.	43	43 (100%)	Multiple	10–113	CAPTURE	*S. avermitilis* SUKA17 *S. lividans* TK24 *B. subtilis* JH642	7 (16%)	5	(Enghiad et al., 2021)
100 *Streptomyces spp.*	Orphan PKS, NRPS, PKS-NRPS	58 (72%)	PKS, NRPS	140	Random library	*S. albus* J1074 *S. lividans* RedStrep 1.7	15 (24%)	3	(Libis et al., 2022)
1 Bacteroidota 10 Pseudomonadota 3 Cyanobacteriota 5 Actinomycetota 8 Bacillota	96	83 (86%)	RiPPs	<18	Golden Gate assembly of synthetic genes	*E. coli* BL21 (DE3)	27 (32%)	3	(Ayikpoe et al., 2022)

## BGC Prioritization, Biosynthetic Class, and Host Taxa

The first step in genome mining is BGC selection, which includes the rationale for the selected BGC and the identification of the genes to be cloned. To this end, databases containing information about known BGCs such as MIBiG (Terlouw et al., 2023) are very helpful in the race for novel chemistry. However, they remain incomplete because most known NPs have not yet been connected to their BGCs.

Compared to activation of orphan BGCs in their native host, identification of the complete set of genes necessary for NP biosynthesis in a heterologous host is more challenging because (a) genes may be necessary that are not part of the cluster, and (b) cluster boundaries may not be accurately predicted. Detailed information about the principles underlining current bioinformatic tools for BGC prediction can be found in reference (Z. Xu et al., 2023).

For the studies reviewed here, the rationale for BGC selection was based either on (a) predicted structural novelty, (b) biosynthetic class, (c) structure similarity to known antibiotic class, or (d) biological activity of library clone (Fig. 2A). The most frequent approach (56%) was to prioritize novelty by expressing unusual BGCs found in rare and/or understudied bacteria, exemplified by the discovery of the lanthipeptide marinsedin from *Marinicella sedimis* (Han et al., 2022), and by the discovery of 31 other compounds listed in Supplemental Table S1 (Alberti et al., 2019; Bothwell et al., 2021; Cheng et al., 2023; De Rond et al., 2021; Enghiad et al., 2021; Gao et al., 2023; Gummerlich et al., 2020; Hao et al., 2019; Hashimoto et al., 2021; Kaweewan et al., 2021; Lasch et al., 2021; Lasch, Gummerlich, et al., 2020, 2020; Li et al., 2020; S. H. Liu et al., 2019; Y. Liu et al., 2021; Myronovskyi et al., 2018; Paulus et al., 2022; Shi et al., 2019; Shuai et al., 2020; Unno et al., 2020; Vermeulen et al., 2022; X. Wang et al., 2023; Yuet et al., 2020; Y. Zhang et al., 2021; Y. Zhang et al., 2022).

Eighteen studies (36%) prioritized the expression of a NP class or subclass. For instance, Libis *et al.* targeted adenylation and ketosynthase domains in a genomic library generated from 100 *Streptomyces* strains to identify nonribosomal peptide synthetase (NRPS) and polyketide synthase (PKS) BGCs (Libis et al., 2022). Ayikpoe *et al*. used the bioinformatic tool RODEO (Tietz et al., 2017) to mine phylogenetic diverse bacteria to discover ribosomally synthesized and post translationally modified peptides (RiPPs) (Ayikpoe et al., 2022). Ren *et al*. used RODEO and RRE-Finder (Kloosterman et al., 2020) to uncover a new RiPP subclass (Ren et al., 2023). Several other studies focused on RiPP subclasses such as lasso peptides (Cao et al., 2021; Carson et al., 2023; Cheung-Lee et al., 2019; Cheung‐Lee et al., 2020; Gomez-Escribano et al., 2019; Mevaere et al., 2018), lanthipeptides (Arias-Orozco et al., 2021; Kaweewan et al., 2023; Singh et al., 2020; Thetsana et al., 2022), and thiopeptides (Santos-Aberturas et al., 2019). For further examples, see Supplemental Table S1 (Bösch et al., 2020; J. Liu et al., 2019; Nguyen et al., 2022; Shi et al., 2021).

The third prioritization criterion was based on structure similarity to known antibiotic classes (6%). For example, cadasides and malacidins calcium dependent antibiotics were discovered by searching for NRPS adenylation domains similar to those encoding known calcium dependent antibiotics (Hover et al., 2018; Wu et al., 2019). Likewise, the BGCs encoding glycopeptides GP1416 and GP6738 were selected for expression because of their sequence similarities to known glycopeptide antibiotic BGCs (M. Xu et al., 2020b). Finally, one study used activity-guided prioritization of library clones. A bacterial artificial chromosome (BAC) library of a *Streptomyces rochei* strain was generated, expressed in *Streptomyces lividans* and active clones were prioritized leading to the discovery of a lanthipeptide (M. Xu et al., 2020a).

In summary, over the last 5 years, at least 63 NP families were discovered and characterized using heterologous expression and reported in 50 studies. A family is here defined as structurally similar NPs isolated after the heterologous expression of a singular BGC. Most of the studies reviewed here focused on RiPPs (48%), followed by PKSs, NRPSs, and hybrid PKS-NRPSs (32%), other biosynthetic classes that included terpenoids, alkaloids, and oxazolones (12%), and multiple classes (8%) (Fig. 2B). Most studies (56%) focused on actinomycetes (Fig. 2C). While actinomycetes are indeed gifted (Gavriilidou et al., 2022), mining of diverse phyla is expected to lead to further chemical diversity (Hegemann et al., 2023).

## Cloning Methods

Three general methods were used to clone BGCs of interest for heterologous expression: DNA chemical synthesis or polymerase chain reaction (PCR) followed by assembly, direct cloning from genomic DNA, and generation of random libraries (Fig. 3). Various assembly methods were used to construct plasmids for expression, to perform promoter engineering, or to obtain complete BGCs from library clones. Information about library generation, direct cloning, and assembly methods can be found in references (Huo et al., 2019; W. Wang et al., 2021). Briefly, random libraries are collections of genomic DNA generated by fragmentation of the DNA followed by cloning of the DNA fragments in BAC or cosmid vectors. Direct cloning involves cloning the BGC of interest directly from genomic DNA but not using PCR. Instead, methods such as transformation associated recombination (TAR) cloning or Cas12a-assisted precise targeted cloning using *in vivo* DNA circularization (CAPTURE) are used. Assembly methods include restriction enzyme mediated methods such as Golden Gate assembly, and recombination-based methods such as TAR cloning in yeast, Red/ET-mediated recombineering in bacteria, and *in vitro* Gibson assembly (Huo et al., 2019; W. Wang et al., 2021).

DNA synthesis is advantageous for simplicity, if mutations are to be introduced, or if codon optimization is required. However, synthesis is practical only for small BGCs such as RiPPs since the maximum size that DNA synthesis suppliers currently offer is 1.8 kb for fragments and 5 kb for clonal DNA. Accordingly, DNA synthesis or PCR to amplify the BGC from genomic DNA was used in 87% of the studies expressing only RiPPs (Arias-Orozco et al., 2021; Ayikpoe et al., 2022; Bösch et al., 2020; Bothwell et al., 2021; Cao et al., 2021; Carson et al., 2023; Cheung-Lee et al., 2019; Cheung‐Lee et al., 2020; Gomez-Escribano et al., 2019; Kaweewan et al., 2021, 2023; Koos & Link, 2019; Mevaere et al., 2018; Nguyen et al., 2022; Singh et al., 2020; Thetsana et al., 2022; Unno et al., 2020; Vermeulen et al., 2022; X. Wang et al., 2023; Y. Zhang et al., 2022). TAR cloning, CAPTURE, and random library generation were used in the remaining studies (Ren et al., 2023; Santos-Aberturas et al., 2019; M. Xu et al., 2020a) (Fig. 3).

In contrast, random libraries and direct cloning techniques are often used for cloning of PKS, NRPS, and PKS-NRPS hybrids in line with the larger size of the BGCs compared to RiPPs. In fact, 88% of the studies aiming to express only PKS or NRPS used random library generation and or direct cloning, often in combination with assembly methods (Fig. 3) (Alberti et al., 2019; Gao et al., 2023; Hashimoto et al., 2021; Hover et al., 2018; Lasch et al., 2021; Lasch, Gummerlich, et al., 2020, 2020; Libis et al., 2022; S. H. Liu et al., 2019; Y. Liu et al., 2021; Myronovskyi et al., 2018; Paulus et al., 2022; Shi et al., 2019; Wu et al., 2019; M. Xu et al., 2020b). For example, the libraries generated for the discovery of cadasides, malacindins, and miramides produced cosmids with portions of the desired BGCs. The overlapping pieces of the BGCs were then assembled into a BAC by TAR cloning and integrated into the chromosomes of the hosts (Hover et al., 2018; Paulus et al., 2022; Wu et al., 2019). Moreover, recombination methods like Red/ET can be used to introduce a selective marker (Lasch et al., 2021) or insert integration machinery into the vector (Gummerlich et al., 2020). Studies using random libraries used hosts related to the source organism to ensure the presence of regulatory elements necessary for BGC expression since the BGCs were often not refactored, except for two studies that engineered promoters (Gao et al., 2023; Lasch, Gummerlich, et al., 2020).

In summary, while DNA synthesis is convenient for small BGCs, large BGCs are still technically challenging to obtain through synthesis. Random libraries can accommodate large pieces of DNA; however, much effort must be invested in finding clones that cover the whole BGC of interest. For example, Hashimoto *et al*. screened 1 536 BAC clones to identify the one leading to the production of the polyketide JBIR-159 (Hashimoto et al., 2021). Hover *et al*. and Libis *et al*. generated libraries of 25 000 and 60 000 clones, respectively (Hover et al., 2018; Libis et al., 2022). To facilitate analysis of such libraries, Hover *et al*. used barcoded primers and profiled the obtained clones with the bioinformatic platform eSNaPD (environmental Surveyor of Natural Product Diversity) (Hover et al., 2018). Libis *et al*. also used barcoded primers and analyzed their multigenome library with the targeted sequencing workflow CONKAT-seq (co-occurrence network analysis of targeted sequences) that uses co-occurrence patterns in libraries to identify chromosomally clustered domains thus increasing the probability of assembling complete BGCs from complex samples (Libis et al., 2019, 2022). In contrast, direct cloning methods are convenient as they allow targeted cloning of a BGC of interest but some, such as TAR cloning, have the limitation of not being suitable for BGCs that contain repetitive DNA sequences due to unwanted recombination that can result in BGC deletions. Cas-assisted excision from genomic DNA followed by *in vitro* assembly with a vector has been recently used to circumvent the drawbacks of TAR cloning (Enghiad et al., 2021; Montaser & Kelleher, 2020; J.-W. Wang et al., 2015).

## Host Choice

After cloning the BGC of interest, transferring it into a suitable host is necessary for optimal yields. The heterologous host must be genetically tractable and ideally, easy to culture. *Streptomyces* spp. (Actinomycetia) were used as hosts in 54% of the studies (Fig. 2D) matching the source of the BGCs in most cases (Fig. 4A). Indeed, phylogeny relatedness was mentioned by Hao *et al*. and four other studies as the reason for host choice (Hao et al., 2019; Mevaere et al., 2018; Shi et al., 2021; X. Wang et al., 2023; M. Xu et al., 2020b). Phylogeny as a choice is backed up by a recent study showing that the yield of heterologously expressed NPs is often higher when the host is closely related to the native strain (G. Wang et al., 2019), although exceptions exist (J. J. Zhang et al., 2017). Even though not specifically mentioned as reason, strains related to the source organism were used as host for expression in 29 other studies (Alberti et al., 2019; Carson et al., 2023; Cheng et al., 2023; Enghiad et al., 2021; Gao et al., 2023; Gomez-Escribano et al., 2019; Gummerlich et al., 2020; Han et al., 2022; Hashimoto et al., 2021; Kaweewan et al., 2021; Lasch et al., 2021; Lasch, Gummerlich, et al., 2020, 2020; Li et al., 2020; Libis et al., 2022; J. Liu et al., 2019; S. H. Liu et al., 2019; Y. Liu et al., 2021; Myronovskyi et al., 2018; Nguyen et al., 2022; Paulus et al., 2022; Ren et al., 2023; Santos-Aberturas et al., 2019; Shi et al., 2019; Shuai et al., 2020; Thetsana et al., 2022; Unno et al., 2020; Vermeulen et al., 2022; M. Xu et al., 2020a; Y. Zhang et al., 2021). In total, 35 studies (70%) used hosts that fall in the same class as the source BGC strain.

When phylogeny was not taken into consideration, the model organism *Escherichia coli* was used as host (Fig. 2D and Fig. 4A). Twenty studies (40%) used *E. coli* as host (Fig. 2D) (Ayikpoe et al., 2022; Bösch et al., 2020; Bothwell et al., 2021; Cao et al., 2021; Carson et al., 2023; Cheung-Lee et al., 2019; Cheung‐Lee et al., 2020; De Rond et al., 2021; Han et al., 2022; Kaweewan et al., 2021, 2023; Koos & Link, 2019; Nguyen et al., 2022; Singh et al., 2020; Thetsana et al., 2022; Unno et al., 2020; Vermeulen et al., 2022; X. Wang et al., 2023; Yuet et al., 2020; Y. Zhang et al., 2022). All but two (De Rond et al., 2021; Yuet et al., 2020) of these 20 studies expressed RiPP BGCs (Fig. 4B). Codon optimization was performed during the cloning step in three of the studies (Ayikpoe et al., 2022; Carson et al., 2023; Yuet et al., 2020). Moreover, to improve yields of heterologously produced lanthipeptides in *E. coli*, co-expression with tRNA-Glu and glutamyl-tRNA synthetase from the source organisms was used (Bothwell et al., 2021; Kaweewan et al., 2023).

In contrast, 98% of studies exploring PKS, NRPS, and other classes utilized *Streptomyces* hosts (Fig. 4B) matching the source of the BGCs in most cases. Other hosts explored in the covered literature included *Myxococcus xanthus, Lactococcus lactis*, and *Streptococcus mutans. Myxococcus xanthus* was used to express a refactored BGC from the closely related *Sorangiineae* sp. strain MSr11367 (Gao et al., 2023). Arias-Orozco *et al*. chose *L. lactis* as a host to facilitate NP purification given the accumulation of peptides in inclusion bodies (Arias-Orozco et al., 2021). Hao *et al*. developed *S. mutans* UA159 as a host and used it to discover mutanocyclin from human oral bacteria. The natural competence system of *S. mutans* facilitates BGC transfer (Hao et al., 2019).

In summary, *Streptomyces* spp. (Actinomycetia) and *E. coli* (Gammaproteobacteria) were the most frequently used hosts (54% and 40% of studies, respectively). The class of the host matched the source of the expressed BGC in 70% of studies with PKS, NRPS, and hybrid PKS-NRPS BGCs, which were from Actinomycetia and expressed in *Streptomyces* strains, whereas RiPPs from various sources were primarily expressed in *E. coli*.

## Success Rate of Natural Product Discovery by Heterologous Expression

With the increased sophistication of bioinformatic tools and cloning techniques, heterologous expression has become more attractive for NP discovery. From the surveyed literature in the last 5 years, we identified 50 studies reporting NP discovery by heterologous expression. Because only successful attempts are usually reported, it is impossible to predict success rate based on small scale studies. Fortuitously, four large-scale studies from which success rates can be derived were included in the 50 studies reviewed here (Table 1) (Ayikpoe et al., 2022; Enghiad et al., 2021; Gummerlich et al., 2020; Libis et al., 2022). We summarize these four studies below in chronological order of publication.

Gummerlich *et al*. used a random library approach to express BGCs from *Saccharothrix espanaensis* with low similarity to known compounds. Of the 31 BGCs predicted by antiSMASH, six were excluded because they were predicted to encode known NPs or congeners of known NPs. After a BAC library generation, 15 BACs covering 17 of the remaining 25 BGCs were expressed in *S. albus* J1074 and *S. lividans* ΔYA6 strains with 11% expression success rate. Of the four detected products, two were produced in sufficient quantity for isolation. (Gummerlich et al., 2020).

Enghiad *et al*. designed a Cas12a-assisted approach termed CAPTURE for direct cloning of large BGCs from genomic DNA. Briefly, the targeted BGC is digested from genomic DNA with Cas12a, assembled *in vitro* with two DNA receivers containing either an origin of replication or a selection marker, and the generated linear fragment is circularized by Cre-lox recombination *in vivo*. Using CAPTURE, Enghiad *et al*. cloned 43 orphan BGCs from *Streptomyces* spp. and *Bacillus* spp. ranging in size from 10 to 113 kb. (Enghiad et al., 2021). BGCs from *Streptomyces* were expressed in *Streptomyces avermitilis* and *Streptomyces lividans* whereas BGCs from *Bacillus* were introduced in *Bacillus subtilis*. HPLC peaks were observed for seven BGCs (all from *Streptomyces*) giving a 16% success rate for NP detection. Five out of the seven were produced in sufficient quantities for structural characterization. After investigating the expression of BGCs without products, the authors found that 60% of those BGCs had low to no detectable RNA in the culture condition tested. They suggested that further refactoring of the BGCs by promoter engineering, expression of positive regulators or deletion of repressors could improve the success rate (Enghiad et al., 2021).

Next, Libis *et al.* generated a DNA library from 100 pooled *Streptomyces* strains. The generated library with an average insert size of 140 kb was analyzed using a targeted sequence workflow to identify clones with complete BGCs. The authors estimated a 72% cloning success for complete PKS and NRPS BGCs. They then selected 58 orphan BGCs that were expressed *in S. albus* J1074 and *S. lividans RedStrep* 1.7. Fifteen out of the 58 orphan BGCs produced a differential mass spectral feature compared to the control, one of which was a known compound, giving a 24% success rate for NP detection. Three out of the 14 new NPs were isolated for structural characterization (Libis et al., 2022). The authors noted the advantage of expressing the BGCs in different hosts to improve success rate as there was only partial overlap regarding which BGC was expressed in which host (only 36% expressed in both hosts).

Finally, Ayikpoe *et al*. (Ayikpoe et al., 2022) attempted cloning and expression of 96 RiPP BGCs. They used Golden Gate assembly of two to nine synthetic genes into BGCs of <18 kb in size. With up to five genes, the assembly success rate was 100%, decreasing thereafter. Overall, they achieved 86% cloning success with 83 of the 96 RiPPs successfully cloned. During the cloning step, the authors refactored the targeted RiPP BGCs by promoter replacement, *E. coli* codon optimization, and incorporation of a histidine tag (except for the lasso peptides and thiopeptides) to facilitate NP isolation. After expression in *E. coli* BL21, 27 of the 83 cloned BGCs produced mass features corresponding to the modified peptide, giving a 32% success rate. The authors noted that classes of RiPPs for which modifying enzymes have not been reconstituted in *E. coli* were not expressed. Of the 30 peptides detected by mass spectrometry, six were tested for activity, and three bioactive peptides were structurally characterized (Ayikpoe et al., 2022).

Taken together, 11% to 32% of the cloned BGCs were successfully expressed. Ayikpoe *et al.* had the highest success rate at 32%. For the other three studies, further refactoring of the BGCs by promoter exchange could have improved the success rate as indicated by the authors. Moreover, factors such as NP toxicity, codon bias and differential regulatory network may also help explain the low success rate.

## Conclusions and Remaining Challenges

From January 2018 to June 2023, at least 50 studies were published that used heterologous expression for NP discovery. Combined, these 50 studies led to the discovery of 63 new families of natural products (Fig. 2). Most of the studies (56%) prioritized BGCs based on structural novelty, followed by biosynthetic class (36%). The main biosynthetic classes prioritized were RiPPs (48%) and PKS, NRPS, and hybrids thereof (32%). This trend reflects the bioinformatics tools available that are tailored to these classes. In fact, of the 33 tools available to predict BGCs reviewed by Xu *et al*., nine are able to predict only RiPPs, seven are able to predict PKS, NRPS, and hybrid PKS-NRPS BGCs, and the remaining 15 tools can predict multiple types of biosynthetic pathways (Z. Xu et al., 2023). Moreover, RiPPs are more amenable to heterologous expression due to the relatively smaller size of their BGCs compared to PKS and NRPS BGCs.

Actinomycetia genomes were mined most frequently (56%) followed by Gammaproteobacteria (16%). It has been shown that three-fourths of gene cluster families are unique to each bacterial phylum and that diversity drops at each taxonomic rank (Gavriilidou et al., 2022). Thus, exploration of understudied taxa is expected to result in new NPs, expanding the known NP chemical space. However, new approaches will need to be developed to enable access to BGCs from understudied bacteria, such as cultivation conditions to facilitate isolation of specific taxa and a collection of versatile host strains to enable the expression of BGCs from varied sources.

Cloning methods used in the covered literature depended on BGC size, which is correlated with biosynthetic class (Fig. 3). RiPP BGCs tend to be smaller and DNA synthesis or PCR was used most frequently (87%), whereas for large PKS and NRPS BGCs random libraries and direct cloning were the methods of choice. Newer cloning methods are being developed, such as Cas-assisted excision from genomic DNA followed by *in vitro* assembly with a vector, which has been used to circumvent the drawbacks of homologous recombination-based direct cloning (Enghiad et al., 2021; Montaser & Kelleher, 2020; J.-W. Wang et al., 2015).

The main hosts (Fig. 2D) used were *Streptomyces* spp. (54%) and *E. coli* (40%). Most studies (70%) used a host that falls in the same class as the source BGC strain. When phylogeny was not taken into consideration, *E. coli* was the host of choice (Fig. 4A). There was a correlation between host choice and biosynthetic class. For example, 90% of the studies expressing RiPPs from various sources used *E. coli* as host, whereas 98% of the studies targeting PKS, NRPS, and other classes used *Streptomyces* spp. matching the source of the BGCs in most cases (Fig. 4B).

A common question with heterologous expression is whether the heterologously identified compounds are the ones that would have been detected in the native producers. This question cannot always be answered because the biosynthetic gene cluster may be unexpressed in the native host. In fact, eight studies used heterologous expression because they were unable to find the NP associated to their BGC of interest in the native host (Bösch et al., 2020; Bothwell et al., 2021; Cheng et al., 2023; Han et al., 2022; Li et al., 2020; J. Liu et al., 2019; Shi et al., 2019; X. Wang et al., 2023). Only two other of the covered papers were indeed able to identify the heterologous products in the native producers (Gao et al., 2023; J. Liu et al., 2019). Conversely, fralnimycin, isolated after expression of an aminocyclitol-like BGC from *Frankia alni* in *S. albus* Del14 likely derives from the incorporation of a precursor from the host. Fralnimycin is biosynthesized via esterification of salicylic acid and tryptophol. However, no genes responsible for the biosynthesis of tryptophol were found in the heterologously expressed fosmid (Myronovskyi et al., 2018).

Heterologous expression is made possible by bioinformatics tools for genome mining, biosynthetic knowledge base, improved cloning techniques, host development, improved isolation, and detection of the desired NP (Avalon et al., 2022; Caesar et al., 2021; Huo et al., 2019; J. Liu et al., 2020) (Fig. 1). The studies covered here support heterologous expression as a promising way to access novel chemistry. In particular, the recently reported large-scale studies are significant and instrumental in revealing current limitations for the field to address. The low success rate of large-scale studies makes it apparent that much remains to be improved to allow us to seamlessly go from DNA to natural products using heterologous expression.

The reasons for failure to be considered are numerous. Ayikpoe *et al*. considered three factors that can lead to failure and addressed those factors with their design, i.e., gene expression, product toxicity, and product purification, which likely explain their higher success rate compared to the other large-scale studies (Table 1). First, gene expression was addressed with codon optimization for the host of choice, and the synthetic genes were placed under a promoter known to work well in the host. Second, toxicity was avoided by producing inactive precursors that were then converted to the final products *in vitro*. Third, purification was facilitated by inserting a His-tag. Their design may explain the higher success rate compared to the other studies. Yet, biosynthetic class also plays a role as some of the strategies used are applicable to RiPPs but not to other classes. For example, the strategy to circumvent potential toxicity was to exclude the protease gene because products containing the leader peptide are expected to be inactive. *In vitro* enzymatic cleavage of the leader peptides was then used to obtain the final products. This strategy also allowed the insertion of a His-Tag at the *N*-terminus of the precursor peptide, which can then be removed during leader peptide cleavage. This approach is very elegant and works well for RiPPs (with lasso peptides and thiopeptides as exceptions) but not for other biosynthetic classes. Including resistance genes is an alternative strategy that is applicable to all biosynthetic classes, if resistance genes are associated with the BGC. Incidentally, resistance genes can also be used as a prioritization rationale to identify antibiotics (Yan et al., 2020).

We next discuss four overall factors that can affect success, some of which are easier to address than others, that is, gene expression, host choice, incomplete BGCs, and sequence errors.

Insufficient gene expression is a major hurdle. Gene expression can be improved with refactoring using well-characterized promoters and codon optimization, as done by Ayikpoe *et al*. However, refactoring alone is not sufficient as evidenced by the 32% success rate. Because different bacteria have different codon preferences, it is assumed that optimizing the sequence by using codons that are common in the host, improves expression. However, codon optimization is not always successful (Jenkins et al., 2023). Moreover, while protein yield may be improved with codon optimization, protein function may be reduced (Arsın et al., 2021). It has been shown that many genes contain rare codons interspaced among common codons and that rare codons are important to slow down translation and allow proper protein folding (Y. Liu, 2020). Thus, *codon harmonization*—reproducing the order of common and rare codons for the host of interest—can lead to improved enzyme activity (Angov et al., 2008; Arsın et al., 2021; Y. Liu, 2020; Mignon et al., 2018). A new open source tool for codon optimization/harmonization became available during revision of this article (Schmidt et al., 2023). We also speculate that any recoding (even codon harmonization) may lead to detrimental, context-dependent transcription and translation effects (Biziaev et al., 2022; Jiang et al., 2023; Kent et al., 2018) that cannot yet be predicted and avoided.

With a host that is phylogenetically related to the source DNA, codon optimization/harmonization can be avoided, and regulatory elements are expected to be better recognized by the host. In fact, 70% of the studies selected phylogenetically related hosts (Fig. 4A). By expressing nine BGCs in 25 hosts, Wang *et al*. (G. Wang et al., 2019) demonstrated that success rate in terms of yield and number of congeners detected tends to improve with increasing relatedness (defined as 16S rRNA sequence identity) between source and host. Yet, some versatile host strains were identified that had higher success rates (produced more natural products), outperforming others despite lower relatedness. Thus, host selection is an important part of heterologous expression pipelines. It seems that the highest success rates would be achieved when using many host strains rather than only one. Because increasing the number of hosts increases complexity and costs, it is then advantageous to select and develop several versatile strains to be added to the heterologous expression toolbox. Versatile strains may also help address other potential reasons for failure, such as missing precursors and incorrect folding of proteins.

Addressing incomplete BGCs remains a challenge because of split clusters and because determination of the boundaries requires experimental validation. To help define the DNA region to be cloned, algorithms have been developed for automatic prediction of cluster boundaries (Blin et al., 2017) and boundaries can be estimated by comparing BGCs from multiple strains (Adamek et al., 2017). For rare and unusual BGCs, estimating the boundaries becomes more difficult. In this respect, being generous regarding the number of surrounding genes to be included can pay off. At the same time, increasing the size of the predicted BGC also makes cloning more difficult. Finally, for synthetic DNA, any errors in the original sequence data will carry over into synthetic constructs; thus, the quality of the source genome sequence is important.

Given all the potential reasons for failure, some of which are difficult to address such as incomplete BGCs, aiming for 100% heterologous expression success rate for unknown BGCs appears unrealistic as of 2023. Yet, the studies reviewed here point to a combination of approaches that should be used to improve the chances of success, including promoter replacement to ensure transcription, addressing product toxicity, and testing various, versatile hosts. We expect these combined approaches may help double the current best success rate of 32%. In fact, by testing different hosts, Wang *et al*. (G. Wang et al., 2019) reached 67% success rate. Other yet unknown or unexplored factors may help improve the success rate further in the future.

## Supplementary Material

kuad044_Supplemental_File
