# Supplementary material for: Bacterial natural product discovery by heterologous expression
Source: J Ind Microbiol Biotechnol. 2023 Dec 5;50(1):kuad044. doi: 10.1093/jimb/kuad044 (PMC10727000; doi:10.1093/jimb/kuad044)
Supplement: kuad044_Supplemental_File [file kuad044_supplemental_file.pdf]

Supplemental information

Bacterial natural product discovery by heterologous expression

A. Elodie Kadjo,[a,b] Alessandra S. Eustáquio[a,b*]

[a]Department of Pharmaceutical Sciences, College of Pharmacy, University of Illinois at Chicago, Chicago, IL, 60607, USA

[b]Center for Biomolecular Sciences, College of Pharmacy, University of Illinois at Chicago, Chicago, IL 60607, USA

SI Table S1. Summary of reviewed studies.

Biosynthetic class	Rationale for BGC prioritization	Cloning method	BGC Source	Host choice	Source taxa (class)	Host taxa (class)	Natural Products	Ref.
PKS, NRPS, hybrid thereof	Biosynthetic class	Library	100 *Streptomyces strains*	*S. albus* J1074, *S. lividans* RedStrep 1.7	Actinomycetia	Actinomycetia	Prolinolexin, Cinnamexin, Conkatamycin	(Libis et al. 2022)
Multiple classes	Structural novelty	Assembly	14 *Streptomyces* and 3 *Bacillus*	*S. avermitilis* SUKA17, S. *lividans* TK24, *B. subtilis* JH642	Actinomycetia	Actinomycetia	Bipentaromycins, Allenomycins, Angucyclines, Anthraquinones	(Enghiad et al. 2021)
RiPP	Structural novelty	Synthesis & Assembly	*Marinicella sediminis* F2T	*E. coli* BL21 (DE3)	Gamma-proteobacteria	Gamma-proteobacteria	Marinsedin	(Han et al. 2022)
PKS, NRPS, hybrid thereof	Structural novelty	Library	*S. versipellis* 4083-SVS6	*S. lividans* TK23	Actinomycetia	Actinomycetia	Polyene compound JBIR-159	(Hashimoto et al. 2021)
Multiple classes	Structural novelty	Assembly	*Streptococcus* strains, *Staphylococcus* strain, *Clostridium* strain	*Streptococcus mutans* UA159	Bacilli	Bacilli	Lipopeptide SNC1-465	(Hao et al. 2019)
RiPP	Structural novelty	Synthesis & Assembly	*Bacillus amyloliquefaciens*	*E. coli* BL21 (DE3)	Bacilli	Gamma-proteobacteria	Amylopeptins	(Zang et al 2021)
PKS, NRPS, hybrid thereof	Structural novelty	Library & Assembly	*S. albus ssp. chlorinus* NRRL B-24108	*S. albus* Del14	Actinomycetia	Actinomycetia	Bosecamin	(Lasch et al. 2021)

PKS, NRPS, hybrid thereof	Structural novelty	Library	*S. sp.* NA03103	*S. lividans* SBT18	Actinomycetia	Actinomycetia	Ashimides	(Shi et al. 2019)
PKS, NRPS, hybrid thereof	Structural novelty	Synthesis & Assembly	*Nocardia* strains associated with nocardiosis	*E. coli*	Actinomycetia	Gamma-proteobacteria	NOCAP (nocardis associated polyketide)	(Yuet et al. 2020)
Other	Structural novelty	Synthesis & Assembly	*Pseudoalteromonas rubra, Rheinheimera pacifica, Colwellia chukchiensis, Skermanella aerolata, Undibacterium pigrum*	*E. coli*	Many	Gamma-proteobacteria	Tyrazolones, Phenazolones	(De Rond, Asay, and Moore 2021)
RiPP	Structural novelty	Synthesis & Assembly	*Pseudoalteromonas flavipulchra* S16	*E. coli* BL21 (DE3)	Gamma-proteobacteria	Gamma-proteobacteria	Pseudorosins	(Wang et al. 2023)
PKS, NRPS, hybrid thereof	Structural novelty	Library	*S. sp.* B59	*S. albus* J1074	Actinomycetia	Actinomycetia	Shuangdaolides	(Y. Liu et al. 2021)
PKS, NRPS, hybrid thereof	Structural novelty	Library & Assembly	*S. albus ssp. Chlorinus* NRRL B-24108	*S. albus* Del14, *S. lividans* Del8	Actinomycetia	Actinomycetia	Dudomycins	(Lasch, Stierhof, et al. 2020)

Other	Structural novelty	Synthesis & Assembly	*S. venezuelae* ATCC 15439	*S. venezuelae, S. coelicolor*	Actinomycetia	Actinomycetia	Venezuelaenes	(Li et al. 2020)
PKS, NRPS, hybrid thereof	Structural novelty	Library	*Frankia alni* strain ACN14a	*S. albus* Del14	Actinomycetia	Actinomycetia	Fralnimycin	(Myronovskyi et al. 2018)
PKS, NRPS, hybrid thereof	Structural novelty	Library & Assembly	*Sorangiineae* strain MSr11367	*Myxococcus xanthus* DK1622	Myxococcia	Myxococcia	Sorangibactin	(Gao et al. 2023)
Other	Structural novelty	Library	*Micromonospora sp.* SCSIO 07395 (actinomycete)	*S. albus* J1074, *S. albus* del14	Actinomycetia	Actinomycetia	Microechmycins	(Cheng et al. 2023)
RiPP	Structural novelty	Synthesis & Assembly	*Thermobifida fusca*	*E. coli* BL21	Actinomycetia	Gamma-proteobacteria	Fuscanodin	(Koos and Link 2019)
RiPP	Structural novelty	Synthesis & Assembly	*Marinomonas fungiae* strain JCM 18476 T	*E. coli* BL21 (DE3)	Gamma-proteobacteria	Gamma-proteobacteria	Marinomonasin	(Kaweewan, Nakagawa, and Kodani 2021)
PKS, NRPS, hybrid thereof	Structural novelty	Library	*S. seoulensis* A01	*S. lividans* SBT18, *S. coelicolor* M1146, *S. albus* J1074	Actinomycetia	Actinomycetia	Ansaseomycin	(S. H. Liu et al. 2019)
Other	Structural novelty	Library & Assembly	*Kutzneria albida* DSM 43870	*S. albus* Del14	Actinomycetia	Actinomycetia	Huimycin	(Shuai et al. 2020)

PKS, NRPS, hybrid thereof	Structural novelty	Library & Assembly	*Micromonospora endolithica*	*S. albus* Del14	Actinomycetia	Actinomycetia	Loseolamycins	(Lasch, Gummerlich, et al. 2020)
PKS, NRPS, hybrid thereof	Structural novelty	Library & Assembly	*S. mirabilis* Lu17588	*S. albus* Del14	Actinomycetia	Actinomycetia	Miramides	(Paulus et al. 2022)
RiPP	Structural novelty	Synthesis & Assembly	*Pedobacter lusitanus* NL19	*E. coli*	Sphingobacteriia	Gamma-proteobacteria	PedAs	(Bothwell et al. 2021)
Multiple classes	Structural novelty	Library & Assembly	*Saccharothrix espanaensis*	S. lividans DYA6, *S. albus* J1074	Actinomycetia	Actinomycetia	Pentangumycin, SEK90	(Gummerlich et al. 2020)
PKS, NRPS, hybrid thereof	Structural novelty	Assembly	*S. sclerotialus* NRRL ISP-5269	*S. albus* J1074, *S. coelicolor* M1152	Actinomycetia	Actinomycetia	Scleric acid	(Alberti et al. 2019)
RiPP	Structural novelty	Synthesis & Assembly	*Thalassomonas viridans* XOM25T	*E. coli*	Gamma-proteobacteria	Gamma-proteobacteria	VsdsAs	(Vermeulen et al. 2022)
Other	Structural novelty	Assembly	*S. leeuwenhoekii* NRRL B-24963	*S. coelicolor* M1146, *S. albus* J1074	Actinomycetia	Actinomycetia	Streptoazines	(Zhang et al. 2021)
RiPP	Structural novelty	Synthesis & Assembly	*Grimontia marina*	*E. coli* BL21 (DE3)	Gamma-proteobacteria	Gamma-proteobacteria	Grimoviridin	(Unno et al. 2020)
PKS, NRPS, hybrid thereof	Biosynthetic class	Assembly	*S. chrestomyceticus*	*S. lividans* TK24	Actinomycetia	Actinomycetia	Guanipiperazines	(Shi et al. 2021)

Other	Biosynthetic class	Synthesis & Assembly	*S. monomycini*	*S. coelicolor* M1146	Actinomycetia	Actinomycetia	Guanitrypmycins	(Liu et al. 2019)
RiPP	Biosynthetic class	Synthesis & Assembly	Bacteroidota, Pseudomonadota, Cyanobacteriota, Actinomycetota and Bacillota	*E. coli* BL21 (DE3)	Many	Gamma-proteobacteria	LanII, LanII-2A, LanII-2B	(Ayikpoe et al. 2022)
RiPP	Biosynthetic class	Assembly	*Microbacterium paraoxydans* DSM 15019	*S. lividans* TK24, *S. albus* J1074.	Actinomycetia	Actinomycetia	Daptides	(Ren et al. 2023)
RiPP	Biosynthetic class	Synthesis & Assembly	*Methylovulum psychrotolerans*	*E. coli*	Gamma-proteobacteria	Gamma-proteobacteria	Mpr RiPPs	(Nguyen et al. 2022)
RiPP	Biosynthetic class	Synthesis & Assembly	*Cyanobacterium Kamptonema* sp. PCC 6506	*E. coli* BL21 (DE3)	Cyanophyceae	Gamma-proteobacteria	Landornamides	(Bösch et al. 2020)
RiPP	Biosynthetic class	Synthesis & Assembly	*Actinoalloteichus fjordicus* ADI127-1, *S. sp.* ADI94-01, *S. sp.* ADI98-10, *S. noursei* ATCC 11455, *S. venezuelae* ATCC 10712	*S. albus* J1074, *S. lividans* TK24	Actinomycetia	Actinomycetia	9401_LP1, 9810_LP, Snou_LP	(Mevaere et al. 2018)
RiPP	Biosynthetic class	Synthesis & Assembly	*Enterobacter cloacae* complex	*E. coli*	Gamma-proteobacteria	Gamma-proteobacteria	Cloacaenodin	(Carson et al. 2023)
RiPP	Biosynthetic class	Synthesis & Assembly	*Burkholderia ubonensis* MSMB2207	*E. coli* BL21	Beta proteobacteria	Gamma-proteobacteria	Ubonodin	(Cheung-Lee et al. 2020)

RiPP	Biosynthetic class	Synthesis & Assembly	*Pandoraea norimbergensis*	*E. coli* BL21	Beta proteobacteria	Gamma-proteobacteria	Pandonodin	(Cheung-Lee, Cao, and Link 2019)
RiPP	Biosynthetic class	Synthesis & Assembly	*S. leeuwenhoekii* C34T	*S. coelicolor* M1152, *S. coelicolor* M1154	Actinomycetia	Actinomycetia	Leepeptin	(Gomez-Escribano et al. 2019)
RiPP	Biosynthetic class	Synthesis & Assembly	*Thermobifida cellulosilytica, Lihuaxuella thermophila*	*E. coli* BL21 (DE3)	Many	Gamma-proteobacteria	Cellulonodin-2, Lihuanodin	(Cao et al. 2021)
RiPP	Biosynthetic class	Synthesis & Assembly	*Synechococcus* MITS9509	*Lactococcus lactis* NZ9000	Cyanophyceae	Bacilli	SyncAs	(Arias-Orozco et al. 2021)
RiPP	Biosynthetic class	Synthesis & Assembly	*Thermosporothrix hazakensis*	*E. coli* BL21 (DE3)	Ktedonobacteria	Gamma-proteobacteria	Hazakensins	(Kaweewan et al. 2023)
RiPP	Biosynthetic class	Synthesis & Assembly	*S. roseosprus* NRRL 11379	*E. coli* BL21 (DE3)	Actinomycetia	Gamma-proteobacteria	Roseocins	(Singh et al. 2019)
RiPP	Biosynthetic class	Synthesis & Assembly	*Thalassomonas actiniarum*	*E. coli* BL21 (DE3)	Gamma-proteobacteria	Gamma-proteobacteria	Thalassomonasins	(Thetsana et al. 2022)
RiPP	Biosynthetic class	Assembly	*S. varsoviensis*	*S. lividans* TK24, *S. coelicolor* M1146, *S. coelicolor* M1152	Actinomycetia	Actinomycetia	Thiovarsolins	(Santos-Aberturas et al. 2019)
PKS, NRPS, hybrid thereof	Structural similarity to known NP	Library & Assembly	Soil metagenome	*S. albus* J1074	Unknown	Actinomycetia	Cadasides	(Wu et al. 2019)

PKS, NRPS, hybrid thereof	Structural similarity to known NP	Library & Assembly	Soil metagenome	*S. albus* J1074	Unknown	Actinomycetia	Malacidins	(Hover et al. 2018)
PKS, NRPS, hybrid thereof	Structural similarity to known NP	Assembly	*Amycolatopsis sp.* WAC01416 and 7 *Streptomyces* strain	*S. coelicolor*	Actinomycetia	Actinomycetia	GP6738, GP6738	(Xu et al. 2020)
RiPP	Activity	Library	*S. rochei* Sal35	*S. lividans* GX28	Actinomycetia	Actinomycetia	Lexapeptide	(Xu et al. 2020)

References

Alberti, Fabrizio, Daniel J. Leng, Ina Wilkening, Lijiang Song, Manuela Tosin, and Christophe Corre. 2019. “Triggering the Expression of a Silent Gene Cluster from Genetically Intractable Bacteria Results in Scleric Acid Discovery.” *Chemical Science* 10 (2): 453–63. https://doi.org/10.1039/C8SC03814G.

Arias-Orozco, Patricia, Maartje Inklaar, Judith Lanooij, Rubén Cebrián, and Oscar P. Kuipers. 2021. “Functional Expression and Characterization of the Highly Promiscuous Lanthipeptide Synthetase SyncM, Enabling the Production of Lanthipeptides with a Broad Range of Ring Topologies.” *ACS Synthetic Biology* 10 (10): 2579–91. https://doi.org/10.1021/acssynbio.1c00224.

Ayikpoe, Richard S., Chengyou Shi, Alexander J. Battiste, Sara M. Eslami, Sangeetha Ramesh, Max A. Simon, Ian R. Bothwell, et al. 2022. “A Scalable Platform to Discover Antimicrobials of Ribosomal Origin.” *Nature Communications* 13 (1): 6135. https://doi.org/10.1038/s41467-022-33890-w.

Bothwell, Ian R., Tânia Caetano, Raymond Sarksian, Sónia Mendo, and Wilfred A. Van Der Donk. 2021. “Structural Analysis of Class I Lanthipeptides from *Pedobacter Lusitanus* NL19 Reveals an Unusual Ring Pattern.” *ACS Chemical Biology* 16 (6): 1019–29. https://doi.org/10.1021/acschembio.1c00106.

Bösch Nina M., Borsa Mariana, Greczmiel Ute, Morinaka Brandon I., Gugger Muriel, Oxenius Annette, Vagstad Anna L., Piel Jörn. 2020. Landornamides: Antiviral Ornithine-Containing Ribosomal Peptides Discovered through Genome Mining *Angewandte Chemie (International ed. in English)*, *59*, 11763. https://doi.org/10.1002/anie.201916321.

Cao, Li, Moshe Beiser, Joseph D. Koos, Margarita Orlova, Hader E. Elashal, Hendrik V. Schröder, and A. James Link. 2021. "Cellulonodin-2 and Lihuanodin: Lasso Peptides with an Aspartimide Post-Translational Modification." *Journal of the American Chemical Society* 143 (30): 11690–702. https://doi.org/10.1021/jacs.1c05017.

Carson, Drew V., Monica Patiño, Hader E. Elashal, Alexis Jaramillo Cartagena, Yi Zhang, Megan E. Whitley, Larry So, et al. 2023. "Cloacaenodin, an Antimicrobial Lasso Peptide with Activity against *Enterobacter*." *ACS Infectious Diseases* 9 (1): 111–21. https://doi.org/10.1021/acsinfecdis.2c00446.

Cheng, Ziqian, Qingbo Zhang, Jing Peng, Xiaoyang Zhao, Liang Ma, Changsheng Zhang, and Yiguang Zhu. 2023. "Genomics-Driven Discovery of Benzoxazole Alkaloids from the Marine-Derived Micromonospora Sp. SCSIO 07395." *Molecules* 28 (2): 821. https://doi.org/10.3390/molecules28020821.

Cheung-Lee, Wai Ling, Li Cao, and A. James Link. 2019. "Pandonodin: A Proteobacterial Lasso Peptide with an Exceptionally Long C-Terminal Tail." *ACS Chemical Biology* 14 (12): 2783–92. https://doi.org/10.1021/acschembio.9b00676.

Cheung-Lee, Wai Ling, Madison E. Parry, Chuhan Zong, Alexis Jaramillo Cartagena, Seth A. Darst, Nancy D. Connell, Riccardo Russo, and A. James Link. 2020. "Discovery of Ubonodin, an Antimicrobial Lasso Peptide Active against Members of the *Burkholderia Cepacia* Complex." *ChemBioChem* 21 (9): 1335–40. https://doi.org/10.1002/cbic.201900707.

De Rond, Tristan, Julia E. Asay, and Bradley S. Moore. 2021. "Co-Occurrence of Enzyme Domains Guides the Discovery of an Oxazolone Synthetase." *Nature Chemical Biology* 17 (7): 794–99. https://doi.org/10.1038/s41589-021-00808-4.

Enghiad, Behnam, Chunshuai Huang, Fang Guo, Guangde Jiang, Bin Wang, S. Kasra Tabatabaei, Teresa A. Martin, and Huimin Zhao. 2021. "Cas12a-Assisted Precise Targeted Cloning Using in Vivo Cre-Lox Recombination." *Nature Communications* 12 (1): 1171. https://doi.org/10.1038/s41467-021-21275-4.

Gao, Yunsheng, Christine Walt, Chantal D. Bader, and Rolf Müller. 2023. "Genome-Guided Discovery of the Myxobacterial Thiolactone-Containing Sorangibactins." *ACS Chemical Biology* 18 (4): 924–32. https://doi.org/10.1021/acschembio.3c00063.

Gomez-Escribano, Juan Pablo, Jean Franco Castro, Valeria Razmilic, Scott A. Jarmusch, Gerhard Saalbach, Rainer Ebel, Marcel Jaspars, Barbara Andrews, Juan A. Asenjo, and Mervyn J. Bibb. 2019. "Heterologous Expression of a Cryptic Gene Cluster from Streptomyces Leeuwenhoekii C34 ^{T} Yields a Novel Lasso Peptide, Leepeptin." Edited by Andrew J. McBain. *Applied and Environmental Microbiology* 85 (23): e01752-19. https://doi.org/10.1128/AEM.01752-19.

Gummerlich, Nils, Yuriy Rebets, Constanze Paulus, Josef Zapp, and Andriy Luzhetskyy. 2020. "Targeted Genome Mining—From Compound Discovery to Biosynthetic Pathway Elucidation." *Microorganisms* 8 (12): 2034. https://doi.org/10.3390/microorganisms8122034.

Han, Yu, Xiaotong Wang, Youming Zhang, and Liujie Huo. 2022. “Discovery and Characterization of Marinsedin, a New Class II Lanthipeptide Derived from Marine Bacterium *Marinicella Sediminis* F2 ^{T}.” *ACS Chemical Biology* 17 (4): 785–90. https://doi.org/10.1021/acschembio.2c00021.

Hao, Tingting, Zhoujie Xie, Min Wang, Liwei Liu, Yuwei Zhang, Weicang Wang, Zhao Zhang, et al. 2019. “An Anaerobic Bacterium Host System for Heterologous Expression of Natural Product Biosynthetic Gene Clusters.” *Nature Communications* 10 (1): 3665. https://doi.org/10.1038/s41467-019-11673-0.

Hashimoto, Takuya, Junko Hashimoto, Noritaka Kagaya, Takehiro Nishimura, Hikaru Suenaga, Makoto Nishiyama, Tomohisa Kuzuyama, and Kazuo Shin-ya. 2021. “A Novel Oxazole-Containing Tetraene Compound, JBIR-159, Produced by Heterologous Expression of the Cryptic Trans-AT Type Polyketide Synthase Biosynthetic Gene Cluster.” *The Journal of Antibiotics* 74 (5): 354–58. https://doi.org/10.1038/s41429-021-00410-9.

Hover, Bradley M., Seong-Hwan Kim, Micah Katz, Zachary Charlop-Powers, Jeremy G. Owen, Melinda A. Ternei, Jeffrey Maniko, et al. 2018. “Culture-Independent Discovery of the Malacidins as Calcium-Dependent Antibiotics with Activity against Multidrug-Resistant Gram-Positive Pathogens.” *Nature Microbiology* 3 (4): 415–22. https://doi.org/10.1038/s41564-018-0110-1.

Kaweewan, Issara, Shinta Ijichi, Hiroyuki Nakagawa, and Shinya Kodani. 2023. “Heterologous Production of New Lanthipeptides Hazakensins A and B Using a Cryptic Gene Cluster of the Thermophilic Bacterium Thermosporothrix Hazakensis.” *World Journal of Microbiology and Biotechnology* 39 (1): 30. https://doi.org/10.1007/s11274-022-03463-6.

Kaweewan, Issara, Hiroyuki Nakagawa, and Shinya Kodani. 2021. “Heterologous Expression of a Cryptic Gene Cluster from Marinomonas Fungiae Affords a Novel Tricyclic Peptide Marinomonasin.” *Applied Microbiology and Biotechnology* 105 (19): 7241–50. https://doi.org/10.1007/s00253-021-11545-y.

Koos, Joseph D., and A. James Link. 2019. “Heterologous and *in Vitro* Reconstitution of Fuscanodin, a Lasso Peptide from *Thermobifida Fusca*.” *Journal of the American Chemical Society* 141 (2): 928–35. https://doi.org/10.1021/jacs.8b10724.

Lasch, Constanze, Nils Gummerlich, Maksym Myronovskyi, Anja Palusczak, Josef Zapp, and Andriy Luzhetskyy. 2020. “Loseolamycins: A Group of New Bioactive Alkylresorcinols Produced after Heterologous Expression of a Type III PKS from Micromonospora Endolithica.” *Molecules* 25 (20): 4594. https://doi.org/10.3390/molecules25204594.

Lasch, Constanze, Marc Stierhof, Marta Rodríguez Estévez, Maksym Myronovskyi, Josef Zapp, and Andriy Luzhetskyy. 2020. “Dudomycins: New Secondary Metabolites Produced after Heterologous Expression of an Nrps Cluster from Streptomyces Albus Ssp. Chlorinus Nrrl B-24108.” *Microorganisms* 8 (11): 1800. https://doi.org/10.3390/microorganisms8111800.

———. 2021. “Bonsecamin: A New Cyclic Pentapeptide Discovered through Heterologous Expression of a Cryptic Gene Cluster.” *Microorganisms* 9 (8): 1640. https://doi.org/10.3390/microorganisms9081640.

Li, Zhong, Yuanyuan Jiang, Xingwang Zhang, Yimin Chang, Shuai Li, Xiaomin Zhang, Shanmin Zheng, et al. 2020. “Fragrant Venezuelaenes A and B with A 5–5–6–7 Tetracyclic Skeleton: Discovery, Biosynthesis, and Mechanisms of Central Catalysts.” *ACS Catalysis* 10 (10): 5846–51. https://doi.org/10.1021/acscatal.0c01575.

Libis, Vincent, Logan W. MacIntyre, Rabia Mehmood, Liliana Guerrero, Melinda A. Ternei, Niv Antonovsky, Ján Burian, Zongqiang Wang, and Sean F. Brady. 2022. “Multiplexed Mobilization and Expression of Biosynthetic Gene Clusters.” *Nature Communications* 13 (1): 5256. https://doi.org/10.1038/s41467-022-32858-0.

Liu, Jing, Xie, Xiulan, Li, Shu-Ming. 2019. Guanitrypmycin Biosynthetic Pathways Imply Cytochrome P450 Mediated Regio- and Stereospecific Guaninyl-Transfer Reactions. *Angewandte Chemie (International ed. in English)*, *58*, 11534. https://doi.org/10.1002/anie.201906891.

Liu, Shuang He, Wen Wang, Kai Biao Wang, Bo Zhang, Wei Li, Jing Shi, Rui Hua Jiao, Ren Xiang Tan, and Hui Ming Ge. 2019. “Heterologous Expression of a Cryptic Giant Type I PKS Gene Cluster Leads to the Production of Ansaseomycin.” *Organic Letters* 21 (10): 3785–88. https://doi.org/10.1021/acs.orglett.9b01237.

Liu, Yang, Haibo Zhou, Qiyao Shen, Guangzhi Dai, Fu Yan, Xiaoju Li, Xiangmei Ren, et al. 2021. “Discovery of Polycyclic Macrolide Shuangdaolides by Heterologous Expression of a Cryptic *Trans* -AT PKS Gene Cluster.” *Organic Letters* 23 (17): 6967–71. https://doi.org/10.1021/acs.orglett.1c02589.

Mevaere, Jimmy, Christophe Goulard, Olha Schneider, Olga N. Sekurova, Haiyan Ma, Séverine Zirah, Carlos Afonso, Sylvie Rebuffat, Sergey B. Zotchev, and Yanyan Li. 2018. “An Orthogonal System for Heterologous Expression of Actinobacterial Lasso Peptides in Streptomyces Hosts.” *Scientific Reports* 8 (1): 8232. https://doi.org/10.1038/s41598-018-26620-0.

Myronovskyi, Maksym, Birgit Rosenkränzer, Suvd Nadmid, Petar Pujic, Philippe Normand, and Andriy Luzhetskyy. 2018. “Generation of a Cluster-Free Streptomyces Albus Chassis Strains for Improved Heterologous Expression of Secondary Metabolite Clusters.” *Metabolic Engineering* 49 (September): 316–24. https://doi.org/10.1016/j.ymben.2018.09.004.

Nguyen, Nguyet A., Ying Cong, Rachel C. Hurrell, Natalie Arias, Neha Garg, Aaron W. Puri, Eric W. Schmidt, and Vinayak Agarwal. 2022. “A Silent Biosynthetic Gene Cluster from a Methanotrophic Bacterium Potentiates Discovery of a Substrate Promiscuous Proteusin Cyclodehydratase.” *ACS Chemical Biology* 17 (6): 1577–85. https://doi.org/10.1021/acschembio.2c00251.

Paulus, Constanze, Maksym Myronovskyi, Josef Zapp, Marta Rodríguez Estévez, Maria Lopatniuk, Birgit Rosenkränzer, Anja Palusczak, and Andriy Luzhetskyy. 2022. “Miramides A–D: Identification of Detoxin-like Depsipeptides after Heterologous Expression of a Hybrid NRPS-PKS Gene Cluster from Streptomyces Mirabilis Lu17588.” *Microorganisms* 10 (9): 1752. https://doi.org/10.3390/microorganisms10091752.

Ren, Hengqian, Shravan R. Dommaraju, Chunshuai Huang, Haiyang Cui, Yuwei Pan, Marko Nesic, Lingyang Zhu, David Sarlah, Douglas A. Mitchell, and Huimin Zhao. 2023. "Genome Mining Unveils a Class of Ribosomal Peptides with Two Amino Termini." *Nature Communications* 14 (1): 1624. https://doi.org/10.1038/s41467-023-37287-1.

Santos-Aberturas, Javier, Govind Chandra, Luca Frattaruolo, Rodney Lacret, Thu H Pham, Natalia M Vior, Tom H Eyles, and Andrew W Truman. 2019. "Uncovering the Unexplored Diversity of Thioamidated Ribosomal Peptides in Actinobacteria Using the RiPPER Genome Mining Tool." *Nucleic Acids Research* 47 (9): 4624–37. https://doi.org/10.1093/nar/gkz192.

Shi, Jing, Xiang Xu, Pei Yi Liu, Yi Ling Hu, Bo Zhang, Rui Hua Jiao, Ghader Bashiri, Ren Xiang Tan, and Hui Ming Ge. 2021. "Discovery and Biosynthesis of Guanipiperazine from a NRPS-like Pathway." *Chemical Science* 12 (8): 2925–30. https://doi.org/10.1039/D0SC06135B.

Shi, Jing, Ying Jie Zeng, Bo Zhang, Fen Li Shao, Yan Chi Chen, Xiang Xu, Yang Sun, Qiang Xu, Ren Xiang Tan, and Hui Ming Ge. 2019. "Comparative Genome Mining and Heterologous Expression of an Orphan NRPS Gene Cluster Direct the Production of Ashimides." *Chemical Science* 10 (10): 3042–48. https://doi.org/10.1039/C8SC05670F.

Shuai, Hui, Maksym Myronovskyi, Suvd Nadmid, and Andriy Luzhetskyy. 2020. "Identification of a Biosynthetic Gene Cluster Responsible for the Production of a New Pyrrolopyrimidine Natural Product—Huimycin." *Biomolecules* 10 (7): 1074. https://doi.org/10.3390/biom10071074.

Singh, M., Chaudhary, S., & Sareen, D. (2020). Roseocin, a novel two-component lantibiotic from an actinomycete. *Molecular microbiology*, *113*(2), 326–337. https://doi.org/10.1111/mmi.14419.

Thetsana, Chanaphat, Shinta Ijichi, Issara Kaweewan, Hiroyuki Nakagawa, and Shinya Kodani. 2022. "Heterologous Expression of a Cryptic Gene Cluster from a Marine Proteobacterium Thalassomonas Actiniarum Affords New Lanthipeptides Thalassomonasins A and B." *Journal of Applied Microbiology* 132 (5): 3629–39. https://doi.org/10.1111/jam.15491.

Unno, Kohta, Issara Kaweewan, Hiroyuki Nakagawa, and Shinya Kodani. 2020. "Heterologous Expression of a Cryptic Gene Cluster from Grimontia Marina Affords a Novel Tricyclic Peptide Grimoviridin." *Applied Microbiology and Biotechnology* 104 (12): 5293–5302. https://doi.org/10.1007/s00253-020-10605-z.

Vermeulen, Ross, Anton Du Preez Van Staden, Leonardo Joaquim Van Zyl, Leon M. T. Dicks, and Marla Trindade. 2022. "Unusual Class I Lanthipeptides from the Marine Bacteria *Thalassomonas Viridans*." *ACS Synthetic Biology* 11 (11): 3608–16. https://doi.org/10.1021/acssynbio.2c00480.

Wang, Xiaotong, Zongjie Wang, Zhiqi Dong, Yihai Yan, Youming Zhang, and Liujie Huo. 2023. "Deciphering the Biosynthesis of Novel Class I Lanthipeptides from Marine *Pseudoalteromonas* Reveals a Dehydratase PsfB with Dethiolation Activity." *ACS Chemical Biology* 18 (5): 1218–27. https://doi.org/10.1021/acschembio.3c00135.

Wu, Changsheng, Zhuo Shang, Christophe Lemetre, Melinda A. Ternei, and Sean F. Brady. 2019. “Cadasides, Calcium-Dependent Acidic Lipopeptides from the Soil Metagenome That Are Active against Multidrug-Resistant Bacteria.” *Journal of the American Chemical Society* 141 (9): 3910–19. https://doi.org/10.1021/jacs.8b12087.

Xu, Min, Wenliang Wang, Nicholas Waglechner, Elizabeth J. Culp, Allison K. Guitor, and Gerard D. Wright. 2020. “GPAHex-A Synthetic Biology Platform for Type IV–V Glycopeptide Antibiotic Production and Discovery.” *Nature Communications* 11 (1): 5232. https://doi.org/10.1038/s41467-020-19138-5.

Xu, M., Zhang, F., Cheng, Z., Bashiri, G., Wang, J., Hong, J., Wang, Y., Xu, L., Chen, X., Huang, S. X., Lin, S., Deng, Z., & Tao, M. (2020). Functional Genome Mining Reveals a Class V Lanthipeptide Containing a d-Amino Acid Introduced by an F_{420} H_2 -Dependent Reductase. *Angewandte Chemie (International ed. in English)*, *59*(41), 18029–18035. https://doi.org/10.1002/anie.202008035.

Yuet, Kai P., Corey W. Liu, Stephen R. Lynch, James Kuo, Wesley Michaels, Robert B. Lee, Abigail E. McShane, Brian L. Zhong, Curt R. Fischer, and Chaitan Khosla. 2020. “Complete Reconstitution and Deorphanization of the 3 MDa Nocardiosis-Associated Polyketide Synthase.” *Journal of the American Chemical Society* 142 (13): 5952–57. https://doi.org/10.1021/jacs.0c00904.

Zhang, Yuqiao, Tingting Yao, Yuechen Jiang, Huayue Li, Weicheng Yuan, and Wenli Li. 2021. “Deciphering a Cyclodipeptide Synthase Pathway Encoding Prenylated Indole Alkaloids in Streptomyces Leeuwenhoekii.” Edited by Haruyuki Atomi. *Applied and Environmental Microbiology* 87 (11): e02525-20. https://doi.org/10.1128/AEM.02525-20.

Zhang, Y., Hong, Z., Zhou, L., Zhang, Z., Tang, T., Guo, E., Zheng, J., Wang, C., Dai, L., Si, T., & Wang, H. (2022). Biosynthesis of Gut-Microbiota-Derived Lantibiotics Reveals a Subgroup of S8 Family Proteases for Class III Leader Removal. *Angewandte Chemie (International ed. in English)*, *61*(6), e202114414. https://doi.org/10.1002/anie.202114414.
